# Evaluation of Infant Injury Prevention Education Provided during Antenatal Classes after Two Years: A Pilot Prospective Cohort Study

**DOI:** 10.3390/ijerph19127195

**Published:** 2022-06-11

**Authors:** Chikako Honda, Kyoko Yoshioka-Maeda, Hitoshi Fujii, Riho Iwasaki-Motegi, Noriko Yamamoto-Mitani

**Affiliations:** 1 Department of Community Health Nursing, Division of Health Sciences and Nursing, Graduate School of Medicine, The University of Tokyo, Tokyo 113-0033, Japan; kyokoy-tky@g.ecc.u-tokyo.ac.jp; 2 Department of Medical Statistics, School of Nursing, Mejiro University, 320 Ukiya, Iwatsuki-ku, Saitama-shi 339-8501, Japan; h.fujii@mejiro.ac.jp; 3 Department of Health Promotion, National Institute of Public Health, 2-3-6, Minami, Wako-shi 351-0197, Japan; motegi.r.ih@niph.go.jp; 4 Department of Gerontological home Care and Long-Term Care Nursing/Palliative Care Nursing, Graduate School of Medicine, The University of Tokyo, Tokyo 113-0033, Japan; noriko-tky@g.ecc.u-tokyo.ac.jp

**Keywords:** antenatal classes, child, group education, infant, injury prevention, mother, pregnancy, program evaluation, prospective study, safety practice

## Abstract

This study examined the long-term effects of an infant injury prevention program implemented during an antenatal class of 131 mothers. Questionnaires were completed 2 years postpartum to assess the incidence of injury (medically attended or home-care), mothers’ perception of injury prevention, implementation of safety practices, and active attitudes toward injury prevention. Responses were obtained from 68 (51.9%) mothers (intervention group, 40; control group, 28), including 24 who reported medically attended injuries and 55 who reported home-care injuries. The incidence of medically attended injuries did not differ between groups. The incidence of home-care injuries was also not significantly different, but was lower in the intervention group (72.5% vs. 92.9%, *p* = 0.050). Significantly fewer children in the intervention group experienced “injury due to being caught between objects” (12.5% vs. 39.3%, *p* = 0.014). Mothers in the intervention group were significantly more aware of injury prevention than those in the control group (*p* = 0.033). The risk of home-care injuries was inversely related to mothers’ injury-prevention perception (odds ratio [OR]: 0.55, *p* = 0.035). This study suggests that group education during pregnancy regarding injury prevention increases mothers’ perception of injury prevention. These findings support implementing injury prevention education during antenatal classes.

## 1. Introduction

Unintentional injuries in children are a significant public health issue, and injury prevention is a global challenge that requires a strategic response [1]. Educating caregivers regarding the safety of children is one of the best critical strategies to prevent unintentional injuries [2]. This strategy increases mothers’ safety-related knowledge and engagement in related behaviors [3] and reduces the overall incidence of injuries, as well as injuries requiring emergency room visits among children [4]. 

A previous study reported that caregiver education according to the developmental stage of the child is crucial [5]. In Japan, infant and child health checkups are provided by the local government at four, 18, and 36 months, resulting in a seamless support system after the birth of the child [6]. During these health checkups, public health nurses (PHNs) educate caregivers regarding injury prevention [7]. 

In spite of this, child injuries continue to occur shortly after birth [8]. Injuries that occur before an infant can turn over are typically preventable via caregiver safety practices (SPs). Therefore, PHNs should provide education to caregivers during pregnancy. Several studies have shown that multiple home visits throughout pregnancy and the early postpartum period increase the use of SPs and decrease injuries to children [3,4,9]. An injury prevention program provided to pregnant women in a nonequivalent control group design trial increased the intention to implement several SPs in the intervention group, and mothers in the intervention group implemented significantly more SPs after birth than those in the control group [10]. However, the long-term effects of one-shot group education in pregnancy remain unclear, and few studies have evaluated if one-shot group education regarding child safety during pregnancy reduces the occurrence of injuries. Determining the effectiveness of such interventions for the prevention of injuries in children will have important implications for future policy and practice.

Therefore, this study examined the long-term effects of an injury prevention educational program for pregnant women. The follow-up questionnaires were completed when the children included in the intervention group reached 2 years of age.

## 2. Materials and Methods

### 2.1. Study Design and Setting

This study is a follow-up to the intervention study in which our injury prevention program was added to an existing antenatal class at a public health center in city X of the Tokyo from November 2017 to June 2018 [10]. The intervention study compared mothers who attended antenatal classes with the program with mothers who attended antenatal classes without the program. The assignment was as follows: mothers who attended antenatal classes from November 2017 to February 2018 (“control months”) were assigned to the control group, and mothers who attended antenatal classes from March to June 2018 (“intervention months”) were assigned to the intervention group. 

The antenatal class consisted of three sessions over 3 weeks, and the injury prevention program was added at the second session. At the first session of the antenatal class, the first author explained the study to the attendees and recruited them, and those who agreed to participate stayed after the second session at their regular antenatal class to attend our group education program on injury prevention that lasted approximately 17 min. The program was designed to remind pregnant women of the importance of having a “safety perspective” (we asked them to imagine what they prioritize when choosing bedding for their baby and to watch a short movie of a baby in action), informing them on common serious injuries and how to prevent infant injuries (suffocation, falls, burns, and accidental ingestion). The program also taught that caregivers should create a safe environment and protect their children. They were encouraged to talk about injury prevention with their families.

This prospective study was conducted when the children of women who participated in that intervention study reached 2 years of age (from December 2019 to October 2020).

### 2.2. Eligibility and Enrollment

The inclusion criteria for our study were: pregnant women expecting their first child, who were in a stable period of pregnancy and could understand the Japanese language. All women attending the antenatal class at the public health center were included in this study, as they met our inclusion criteria [10]. Seventy-five of the antenatal class attendees in the intervention months and 56 of the antenatal class attendees in the control months agreed to participate in the study. The questionnaire was mailed to each participant approximately 1–2 days prior to their child’s second birthday. As the intervention program took place during the second session of the antenatal class, all participants who were absent from the second session were excluded from this study.

### 2.3. Study Outcomes

The primary outcome was the occurrence of childhood injuries between birth and 2 years of age. We asked about all injuries, regardless of whose care and supervision the child was under. We assumed that the intervention with the mother would have a ripple effect on other family members. We also asked about the occurrence of injuries that resulted in a hospital visit (“medically attended injuries”) and that did not (“home-care injuries”). An injury prevention strategy that focuses on more severe injuries could be a one-sided measure that ignores the frequent injuries that more children and caregivers suffer daily [1]. Other questions asked were: the types of injuries, age at which injuries occurred, and causes or circumstances of the injuries. The injuries were classified as having occurred when the child was: 0–4, 5–12, 13–18, or 19–24 months of age. The types of injury were falls, trips, accidental ingestions/foreign bodies, being struck, being caught between objects, burns or scalds, cuts or piercings, drowning, suffocation, and others. These were the top 10 injuries based on emergency-transportation data [11]. 

The secondary outcomes of the study included the mothers’ perceptions of injury prevention, implementation of SPs, and active attitudes toward injury prevention. 

Questionnaire items based on the protective motivation theory [12] and theory of planned behavior [13], which are theories of behavioral change in injury prevention, and the logic model, developed based on interviews with PHNs and specialists during the development of our program [10], were used to assess the mothers’ perceptions of injury prevention. The following four questionnaire items were included: (1) I think serious injuries do not happen to my child (severity and probability of occurrence); (2) children’s safety is something that parents actively create (parental responsibility); (3) it is up to me to keep my child safe (perceived behavioral control); and (4) I try to change/improve my home environment (intention to implement safety practice). A five-point Likert scale ranging from “I strongly agree” to “I do not think so at all” was used, and the answers were rated on a 1–5 scale. 

Questionnaire items regarding the implementation of SPs were developed based on the opinions of pediatric emergency physicians and injury prevention researchers. The following questionnaire items were included: (1) “fall-prevention,” not placing things on the side of the window onto which children could climb; (2) “trip over prevention,” making sure there are no tripping objects or steps around the child; (3) “accidental ingestion-prevention,” small items (such as cigarettes, medicine, and toys) being kept at least one meter above the floor or in a locked cupboard; (4) “burn prevention,” preventing children from touching things that can cause burns, such as irons and rice cookers; (5) “pierce prevention,” not letting children run with pens, forks, or toothbrushes in their mouths; and (6) “drowning prevention,” not leaving a child alone to take a bath or allowing the child to play with water unattended. The SP items were measured using a four-point scale from “always” to “never,” with “always” representing an implementation group and the rest the non-implementation group. 

Questionnaire items based on the logic model created during program development were used to assess the mothers’ active attitudes toward injury prevention. The questionnaire items were as follows. “Have you taken any of the following actions regarding injury prevention in the past 2 years: (1) Have you discussed injury prevention with family and friends? (2) Have you attended a lecture or study session on injury prevention? (3) Have you posted messages about injury prevention on social networking sites?”

The responses were recorded in binary form as “Yes” or “No.” These items were designed to measure the long-term effects of the intervention.

The child’s sex, birth weight, and health conditions that required a hospital visit were also obtained from the questionnaire. Information regarding maternal health conditions that required a hospital visit, subsequent children, maternal educational level, and maternal job status, annual household income, moving after the child’s birth, and smoking within the family, were also recorded. Each participant was given a 1,000-yen library card (worth approximately 8 USD). 

### 2.4. Ethical Considerations

This study was approved by the Ethics Review Board of the University of Tokyo (#11748-[2]) and was conducted according to the Declaration of Helsinki. All study participants provided written informed consent.

### 2.5. Statistical Analysis

The number and proportion of participants who reported injuries (medically attended or home-care) was calculated overall and for each injury type as the primary outcome. A logistic regression analysis was conducted with the data of children who experienced injury or non-injury as the dependent variable and the intervention and control groups as the independent variables. 

The responses to the questionnaire items regarding the perception of injury prevention are reported as mean and standard deviation, and a linear regression analysis was conducted. The number and proportion of participants who answered “must implement” for each item regarding SPs and of those who answered “yes” for each item regarding the active attitude toward injury prevention were determined. A logistic regression analysis was conducted with each item as the dependent variable and the intervention and control groups as the independent variables.

Finally, a multiple logistic regression analysis was performed with injury (medically attended or home-care) as the dependent variable and child and parent basic attributes (child’s sex, mother’s educational background, and household income), perception, and placement in the intervention or control group as independent variables.

All statistical analyses were conducted using Statistical Package for Social Science version 24.0 software (IBM, Armonk, NY, USA). All *p*-values were two-sided. Statistical significance was set at *p* < 0.05. 

## 3. Results

### 3.1. Participant Characteristics and Injury Status up to 24 Months of Age

**Figure 1:**
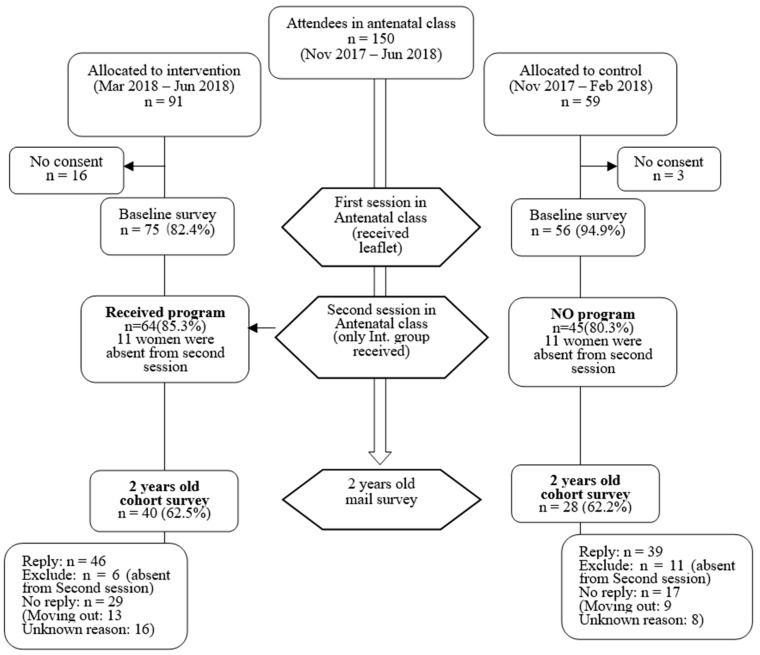
Flowchart of the prospective study.

Of the 131 participants in the baseline study, 68 (51.9%) responded to the follow-up questionnaire (Figure 1).

The participant characteristics were not significantly different between the groups (Table 1). The average birth weight was 2987 g, and 49.2% of participants had male children. More than half of the mothers had a college or higher education (61.8%), and 52.9% had full-time or part-time jobs. Over half (64.2%) of the participants reported an annual household income of ≥7 million yen. 

Overall, 24 children (35.3%) experienced a medically attended injury. The number of children who experienced a medically attended injury was not significantly different between the groups. Fewer children in the intervention group experienced home-care injuries than in the control group, although this difference was not statistically significant (72.5% vs. 92.9%, odds ratio (OR): 0.20, *p* = 0.050). The number of children who were caught between objects was significantly lower in the intervention group than in the control group (12.5% vs. 39.3%, OR: 0.22, *p* = 0.014). “Caught between (objects)” was defined as “an injury caused by being caught between or within objects.” According to the answers, this definition included various cases, such as a parent closing a door without noticing a child’s finger in the door aperture, a finger getting caught in a Japanese-style room fusuma (sliding door), a finger getting caught while trying to close a drawer, and a finger getting caught while opening and closing a window.

### 3.2. Mothers’ Perceptions of Injury Prevention

The mothers in the intervention group demonstrated higher perception scores than those in the control group for all four questions (Table 2). The total perception score was significantly higher in the intervention group (*p* = 0.033).

### 3.3. Implementation Status of SPs and Active Attitudes toward Injury Prevention 

Table 3 shows the implementation status of SPs at home at the time of the survey and the active attitude toward injury prevention during the two years. More than 90% of the participants reported always implementing drowning-prevention SPs, and more than 70% reported always implementing burn-prevention SPs (Table 3). Trip-prevention SPs had the lowest implementation rate (26.5%). There were no significant differences regarding the rate of implementation of any SPs between the two groups. Although there were no significant differences between the two groups regarding the active attitudes of injury prevention, more participants in the intervention group reported talking with family or friends and attending a lecture or study session.

### 3.4. Injury Risk Factors

Female children experienced significantly more medically attended injuries than male children (OR: 4.68, *p* = 0.015) (Table 4). As the perception score increased, the number of home-care injuries decreased (OR: 0.55, *p* = 0.035).

## 4. Discussion

The study showed that providing injury prevention education to pregnant women during an existing antenatal class in a community setting was effective at increasing maternal perceptions of injury prevention. Although heavy-burden interventions such as multiple home visits during pregnancy and the postpartum period have been shown to reduce injuries [4] and increase safe behavior practices and knowledge [3,9], this is the first study addressing the effects of a one-shot intervention on group education for the prevention of childhood injury and on the perception of injury prevention.

In this study, there were no significant differences in medically attended injuries. Home-care injuries were also not significantly different overall, with only “caught between (objects)” injuries being experienced by fewer children in the intervention group. The intervention group had a significantly higher perception of injury prevention than the control group, and mothers with higher perception scores had children who experienced fewer home-care injuries. These results suggest that mothers who received education regarding injury prevention during pregnancy might proactively observe their children’s environment daily and take measures to prevent injuries. The program was structured to: (1) increase pregnant women’s awareness of the importance of adopting a “safety perspective”, (2) teach them about specific safety measures, (3) create a safe environment and protect their children, and (4) encourage them to talk about injury prevention at home. The program might have increased the “safety perspective” and the mothers’ perception of injury prevention. This was indicated by the fact that there were significantly fewer “caught between (objects)” in the intervention group. The risk of these injuries varied from household to household and included door gaps, Japanese-style fusuma (sliding doors), drawers, and windows. Because these factors varied from home to home, there is no end to the list of specific safety measures. However, it is possible that a high perception of injury prevention could have led to a proactive awareness of the risk of injury around the child and a flexible approach to preventing injuries that are more likely to occur, depending on the child’s development. The “caught between (objects)” might have been an injury category wherein differences in parental perception of injury prevention were most likely to emerge.

The “Mothers’ perceptions of injury prevention” also measured perceived behavioral control that “it is up to me to keep my child safe.” The program increased perceived behavioral control and self-efficacy regarding injury prevention behaviors. Pregnancy is a suitable motivation to learn and change health behaviors. The acquisition of knowledge and skills during this period increases self-efficacy, leading to action, and the efficacies of smoking cessation education and obesity prevention guidance have been reported [14,15]. Pregnancy injury-prevention programs may effectively increase caregivers’ perception of injury prevention beyond targeted injuries, allow caregivers to create a safe environment to prevent future injuries and encourage the preventive behaviors of caregivers.

Additionally, mothers’ heightened perceptions of injury prevention might have been passed to their spouses. The program also encouraged mothers to talk to their family members about injury prevention.

We anticipated that the intervention with the mother would have a ripple effect on other family members, such as spouses and grandparents. In the postpartum survey item, “talk about injury prevention with family or friends,” a more significant percentage of the intervention group talked to family or friends (78.3% vs. 58.7%, *p* = 0.044) compared to the non-intervention group. The same question in the present study showed that a larger percentage of the intervention group talked to family or friends, although the difference was not statistically significant (Table 3).

In the postpartum survey, safety practices that could be controlled by the mother alone were promoted, but no significant differences were found for safety practices that others were likely to influence others [10]. For example, “Keep medicines, batteries, and small products in locked cabinets” would be difficult to accomplish if implemented by the mother alone and would require the cooperation of all family members. In future studies, we would consider targeting caregivers other than mothers, such as spouses and grandparents, as direct intervention targets.

The number of children who experienced suffocation, falls, burns, or accidental ingestion was not significantly different between the two groups in this study. While suffocation and burns are two leading causes of infant mortality, the incidence rate is low. In this study, the number of incidents of suffocation and burns was zero; therefore, no analysis regarding these data could be conducted. Falls and accidental ingestion frequently occur in infants and children [16,17]; the risk of their occurrence is well known to parents [18], and they already might have implemented SPs to prevent them, which might have led to the lack of differences between the two groups.

This study showed that female children were significantly more likely than male children to have experienced medically attended injuries. Boys tend to have more frequent and more severe injuries than girls [1], as boys are more prone to risk-taking behaviors [19,20] and parental involvement differs in regards to male and female offspring [21,22]. A previous Japanese study reported no significant difference in the proportion of boy and girl 0–1-year-olds requiring treatment for injuries, though male children were treated for more severe injuries [8]. These results suggest that the parents of female children in this study may have been more conservative regarding injuries and may have more often consulted a doctor for even minor injuries than the parents of male children. In other words, although “medically attended injury” was asked as an indicator of severity, parental perception might have influenced consultation behavior. Thus, using only “medically attended” injuries as an indication of severity may not be the most appropriate way to evaluate injury severity.

The results of this study indicate that PHNs and midwives should provide educational programs regarding injury prevention during pregnancy for first-time parents during antenatal classes. The intervention group in this study retained a high level of perception two years after the education. Parents have more time during pregnancy than the postpartum period to learn about injury prevention [23]. In addition, the opportunity to receive education from professionals during pregnancy is an appropriate time to take responsibility for the safety of children, recognize the need to prevent injuries, and enact behavioral changes. Therefore, PHNs and midwives should consider adopting injury prevention content in the antenatal class, even if only for a short time. 

A resource-consuming program that consisted of a short, one-time, highly sustainable program delivered during existing antenatal classes was developed in this study. A population approach is essential to educate parents regarding common accidents and their prevention. However, the recent spread of infectious diseases renders a group approach difficult [24]. Studies of personalized educational interventions, such as smartphone applications that provide tailor-made individualized prevention programs, have been reported [25]. In the future, the development and implementation of methods to combine group and individual strategies to provide sufficient, detailed information regarding common childhood injuries and prevention methods according to the child’s stage of growth and development are necessary.

This study has limitations. First, the follow-up survey was not designed at the beginning of the intervention study [10]. Therefore, participants were not informed of the follow-up during the initial study, which may have contributed to the low response rate, thus limiting the sample size of the current study. In addition, 16.8% of the participants did not receive the questionnaire as they had moved since the birth of their child. Second, we asked about “medically attended injury” as an indicator of severity of the injury. Although the influence of economic factors is less likely to be a variable due to the availability of universal health coverage through mandatory social health insurance [26], we cannot exclude the possibility that parental perception of the injury may have affected the caregiver’s behavior at hospital visits. Objective indicators are needed, such as asking the mothers to state the specific diagnosis of injury in the future study. Third, recall bias may have influenced the results of the study. Previous studies have shown that the longer the time elapsed and the milder the injury, the less likely it is that parents will recall the accident [27]. For example, parents with a high perception of injury prevention may remember minor injuries, while those with lower perception may underreport injuries. However, our results indicate that parents who had a high perception of injury prevention reported fewer home-care injuries. 

Despite the limitations, this study showed that an educational program during pregnancy increases the caregivers’ perceptions of injury prevention. Future research will include follow-up surveys from the beginning of the intervention to address sample size limitations, devise ways to measure severity scores, and measure outcomes at age one to reduce recall bias. It is also necessary to examine the generalizability of the study by conducting it in other areas. 

## 5. Conclusions

Caregivers who receive the infant-injury prevention program during pregnancy showed a higher perception of injury prevention than those who do not undergo the program. Therefore, injury prevention education effectively increases caregivers’ perceptions and may prevent childhood injuries during the first two years postpartum. The program’s effectiveness should be evaluated in other regions with increased sample sizes to examine the impact on injury occurrence.

## Figures and Tables

**Table 1:** Participant characteristics.

	Total (N = 68)	Intervention (N = 40)	Control (N = 28)	Regression		
**Variables**	**n** **(%)**	**n** **(%)**	**n** **(%)**	**OR**	**(95% CI)**	***p***
**Infant characteristics**						
Sex (male)	30 (49.2)	21 (56.8)	9 (37.5)	0.46	(0.16–1.31)	0.145
Birth weight ≥ 2900 g	34 (54.8)	19 (50.0)	15 (62.5)	0.60	(0.21–1.70)	0.337
Health condition requiring a hospital visit	5 (7.4)	3 (7.7)	2 (7.1)	0.95	(0.15–6.08)	0.956
**Mothers’ characteristics**						
Health condition requiring a hospital visit	9 (13.2)	4 (10.0)	5 (17.9)	1.96	(0.48–8.05)	0.353
Subsequent children	8 (11.8)	2 (5.0)	6 (21.4)	0.19	(0.036–1.04)	0.056
University or graduate school	42 (61.8)	23 (57.5)	19 (67.9)	0.64	(0.23–1.76)	0.388
Full or part-time job	36 (52.9)	21 (52.5)	15 (53.6)	0.96	(0.36–2.52)	0.931
**Home environment**						
Annual household income (thousands of yen) ≥ 7000	43 (64.2)	24 (61.5)	19 (67.9)	0.76	(0.27–2.11)	0.595
Moved after child’s birth	9 (13.2)	5 (12.8)	4 (14.3)	1.17	(0.28–4.77)	0.831
Smoking within the family	14 (20.6)	9 (22.5)	5 (17.9)	0.75	(0.22–2.53)	0.642
**All types of injury (0–24 months)**						
Medically attended	24 (35.3)	13 (32.5)	11 (39.3)	0.74	(0.27–2.04)	0.565
Home-care	55 (80.9)	29 (72.5)	26 (92.9)	0.20	(0.04–1.00)	0.050
**Types of injury (0–24 months)**						
Fall						
Medically attended	9 (13.3)	4 (10.0)	5 (17.9)	0.51	(0.12–2.10)	0.353
Home-care	36 (52.9)	20 (50.0)	16 (57.2)	0.75	(0.28–1.98)	0.562
Trip over						
Medically attended	5 (7.4)	3 (7.5)	2 (7.1)	1.05	(0.16–6.76)	0.956
Home-care	21 (30.9)	11 (27.5)	10 (35.7)	0.68	(0.24–1.93)	0.472
Accidental ingestion/foreign body						
Medically attended	7(10.3)	2 (5.0)	5 (17.9)	0.24	(0.04–1.35)	0.106
Home-care	8 (11.8)	6 (15.0)	2 (7.1)	2.29	(0.43–12.31)	0.333
Struck						
Medically attended	5 (7.4)	3 (7.5)	2 (7.1)	1.05	(0.16–6.76)	0.956
Home-care	18 (26.5)	11 (27.5)	7 (25.0)	1.14	(0.38–3.42)	0.818
Caught between (objects)						
Medically attended ^†^	2 (2.9)	2 (5.0)	0 (0.0)	-	-	-
Home-care	16 (23.5)	5 (12.5)	11 (39.3)	0.22	(0.07–0.73)	0.014
Burn or scald						
Medically attended ^†^	3 (4.4)	3 (7.5)	0 (0.0)	-	-	-
Home-care ^†^	2 (2.9)	0 (0.0)	2 (7.1)	-	-	-
Cut or pierce						
Medically attended ^†^	1 (1.5)	0 (0.0)	1 (3.6)	-	-	-
Home-care	9 (13.3)	5 (12.5)	4 (14.3)	0.86	(0.21–3.52)	0.831
Drowning						
Medically attended ^†^	0 (0.0)	0 (0.0)	0 (0.0)	-	-	-
Home-care	8 (11.8)	3 (7.5)	5 (17.9)	0.37	(0.08–1.71)	0.204
Suffocation						
Medically attended ^†^	0 (0.0)	0 (0.0)	0 (0.0)	-	-	-
Home-care ^†^	0 (0.0)	0 (0.0)	0 (0.0)	-	-	-
Other						
Medically attended ^†^	2 (2.9)	0 0.0	2 (7.1)	-	-	-
Home-care ^†^	0 (0.0)	0 (0.0)	0 (0.0)	-	-	-

Logistic regression analysis: Each variable served as the dependent variable with the intervention or control groups as the independent variables. Abbreviations: CI, confidential interval; OR, odds ratio. ^†^: Could not be tested due to zero participants.

**Table 2:** Mothers’ perceptions of injury prevention.

	Total (N = 68)	Intervention (N = 40)	Control (N = 28)	Regression		
	Mean (SD)	Mean (SD)	Mean (SD)	Β	β	*p*
Severity and probability of occurrence	4.04 (1.17)	4.25 (1.03)	3.75 (1.29)	0.500	0.213	0.081
Parental responsibility	4.34 (0.78)	4.40 (0.67)	4.25 (0.93)	0.150	0.095	0.442
Perceived behavioral control	4.69 (0.62)	4.75 (0.44)	4.61 (0.83)	0.143	0.113	0.361
Intention to implement safety practice	4.25 (0.76)	4.35 (0.62)	4.11 (0.92)	0.243	0.158	0.197
Total score (4–20)	17.38 (2.18)	17.85 (1.4)	16.71 (2.85)	1.136	0.258	0.033

Abbreviations: SD, standard deviation; Β, unstandardized coefficient; β, standardized coefficient.

**Table 3:** Implementation of safety practices and active attitudes regarding injury prevention.

	Total (N = 68) n (%)	Intervention (N = 40) n (%)	Control (N = 28) n (%)	Regression		
				OR	(95%CI)	*p*
**Safety practices**						
Fall prevention	38 (55.9)	19 (47.5)	19 (67.9)	0.43	(0.16–1.17)	0.099
Trip prevention	18 (26.5)	12 (30.0)	6 (21.4)	1.57	(0.52–4.85)	0.432
Accidental-ingestion prevention	36 (52.9)	20 (50.0)	16 (57.1)	0.75	(0.28–1.98)	0.562
Burn prevention	49 (72.1)	31 (77.5)	18 (64.3)	1.91	(0.66–5.59)	0.235
Pierce prevention	41 (60.3)	25 (62.5)	16 (57.1)	1.25	(0.47–3.35)	0.657
Drowning prevention ^†^	63 (92.6)	40 (100.0)	23 (82.1)	-	-	-
**Active attitudes toward injury prevention**						
Talked with family or friends	53 (77.9)	33 (82.5)	20 (71.4)	1.89	(0.59–5.99)	0.282
Attended a lecture or study session	18 (26.5)	12 (30.0)	6 (21.4)	1.57	(0.51–4.85)	0.432
Sent messages on social networking sites	3 (4.4)	1 (2.5)	2 (7.1)	0.33	(0.29–3.87)	0.380

^†^: Could not be tested as 100% of the intervention group implemented this safety practice. Abbreviations: CI, confidential interval; OR, odds ratio.

**Table 4:** Injury risk factors.

	Medically Attended			Home-Care		
	OR	(95% CI)	*p*	OR	(95% CI)	*p*
Sex: Female (Male)	4.68	(1.34–16.30)	0.015	1.44	(0.35–5.88)	0.616
Mother’s educational level: University or Graduate School (Middle or high school)	0.98	(0.28–3.47)	0.976	0.93	(0.21–4.05)	0.920
Household annual income: ≥7000 (<7000)	0.32	(0.09–1.12)	0.075	0.81	(0.17–3.82)	0.794
Perception score	1.17	(0.79–1.73)	0.442	0.55	(0.31–0.96)	0.035
Intervention (control)	0.69	(0.20–2.47)	0.573	0.24	(0.04–1.28)	0.094

The reference categories are shown in parentheses. Missing data were excluded from this analysis. OR, odds ratio; CI, confidential interval. Multivariate logistic regression analysis: medically attended injury/home-care injury = 1, none = 0.

## Data Availability

The data presented in this study are not publicly available because of privacy restrictions.
